# Synchronous Presentation of Type 2 Marine–Lenhart Syndrome and Differentiated Thyroid Carcinoma Manifesting as Thyroid Storm

**DOI:** 10.1155/crie/2498789

**Published:** 2025-08-08

**Authors:** Mennaallah Eid, Kristen Decarlo

**Affiliations:** Division of Endocrinology, Diabetes, and Metabolism, University of Illinois, Chicago, Illinois, USA

## Abstract

Marine–Lenhart syndrome (MLS) is a rare condition characterized by the coexistence of hyperfunctioning thyroid nodules and Graves' disease (GD). The prevalence of thyroid nodules and thyroid cancer is higher in patients with GD. We report a case of 42-year-old female who presented with thyroid storm and found to have underlying GD. An initial thyroid ultrasound (US) revealed two nodules classified as TIRADS 3, whereas a repeat US after achieving euthyroidism, showed changes in the size and consistency of these nodules and identified a new nodule with classification of TIRADS 6 nodule. A 24-h radioactive iodine thyroid uptake scan demonstrated a diffuse increase uptake (75.1%) with one hyperfunctioning and two warm nodules, including the newly identified TIRADS 6 nodule. Fine-needle aspiration (FNA) biopsy confirmed papillary thyroid cancer (Bethesda VI) in a right (R) warm nodule (the TIRADS 6 nodule), while the other two nodules were benign (Bethesda II). The patient underwent a R hemithyroidectomy that was complicated by recurrent laryngeal nerve (RLN) injury. Pathology examination revealed unifocal papillary thyroid microcarcinoma with positive anterior surgical margin. The patient was furtherly treated with radioactive iodine therapy thyroid nodules with GD should be managed cautiously. Emerging evidence challenges the previous notion that hyperthyroidism provides protection against thyroid cancer. The association between GD and thyroid malignancy remains an area of ongoing investigation, with variable management strategies and prognostic implications reported in the literature.

## 1. Introduction

Graves' disease (GD) is the most common cause of hyperthyroidism. The coexistence of autonomous hyperfunctioning thyroid nodules (AFTNs) with GD is called Marine–Lenhart syndrome (MLS) with prevalence of 0.26%–4.1% [1]. It was first described in 1911 [2]. We report a case with Type 2 MLS and differentiated thyroid cancer in a warm nodule.

## 2. Case Presentation

A 42-year-old female with no past medical history presented with agitation, palpitations, diarrhea, and tremors for 2 weeks. On examination, she was agitated and tremulous with arterial blood pressure 180/110 mm of mercury, heart rate 125 beats per min, respiratory rate 26 per min, oxygen saturation 95% on room air, and temperature 101°F. Diffuse thyroid enlargement with a bruit and fine hand tremors were noted. The patient did not have signs of thyroid orbitopathy or pretibial myxedema. Laboratory evaluation confirmed primary hyperthyroidism and positive Graves' and thyroid peroxidase antibodies (Tables 1 and 2). Diagnosis of thyroid storm was made, and the patient was admitted to the intensive care unit. We started her on methimazole 20 mg every 6 h, hydrocortisone 50 mg every 8 h, potassium iodide five drops every 6 h (50 mg/drop, 0.05 mililiter); started 2 h after methimazole, and Esmolol intravenous infusion then oral Propranolol 60 mg every 8 h. Details in Table 1.

**Figure 1:**
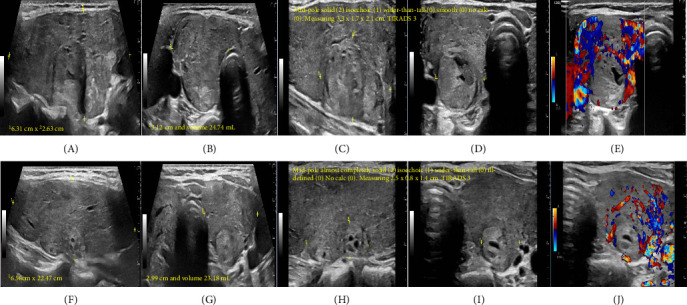
Initial thyroid ultrasound when patient has thyroid storm. (A) Right (R) thyroid lobe in longitudinal axis with heterogenous echogenicity and measuring^1^ 6.31 cm × ^2^2.63 cm. (B) R thyroid lobe in transverse axis with heterogenous echogenicity measuring^1^ width 3.12 cm and volume 24.74 mL. (C) R thyroid mid-pole nodule in longitudinal axis measuring^1^ 3.3 cm × 21.7 cm. (D) R thyroid mid-pole nodule in transverse axis and measuring^1^ 2.1 cm. Mid-pole, solid (2), isoechoic (1), wider-than-tall (0), smooth (0), and no calc (0). Measuring 3.3 cm× 1.7 cm × 2.1 cm. Points: 3. TIRADS 3. (E) R thyroid lobe doppler in transverse axis with hypervascularity. (F) Left (L) thyroid lobe in longitudinal axis with heterogenous echogenicity measuring^1^ 6.56 cm ×^ 2^ 2.47 cm. (G) L thyroid lobe in transverse axis with heterogenous echogenicity and measuring width^1^ 2.99 cm and volume 23.18 mL. (H) L thyroid mid-pole nodule in longitudinal axis and measuring 1 2.5 cm× 2 0.8 cm. (I) L thyroid nodule in transverse axis measuring^1^ 1.4 cm. Mid-pole, Almost completely solid (2,) isoechoic (1), wider-than-tall (0), ill-defined (0), and no calc (0). Measuring 2.5 cm × 0.8 cm × 1.4 cm. Points: 3. TIRADS 3. (J) L thyroid lobe doppler in transverse axis with hypervascularity but less than the R side.

Initial thyroid ultrasound (US) revealed heterogenous thyroid gland enlargement with increased vascularity and two mid-pole thyroid nodules, right (R) nodule measured 3.3 cm and left (L) nodule measured 2.5 cm both described as isoechoic, wider than taller nodules, and classified as TIRADS 3 (Figure 1).

**Figure 2:**
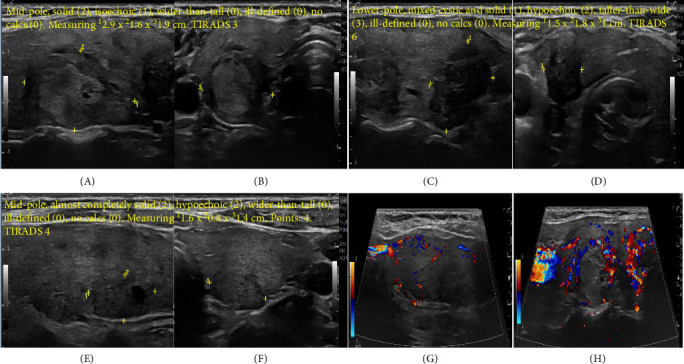
Follow-up thyroid ultrasound after 9 months while patient is clinically and biochemically euthyroid. (A) R mid-pole thyroid nodule in longitudinal axis measuring^1^ 2.94 cm × ^2^ 1.61 cm. (B) R mid-pole thyroid nodule in transverse axis^3^ 1.94 cm. Mid-pole, solid (2), isoechoic (1), wider-than-tall (0), ill-defined (0), and no calcs (0). Measuring 2.9 cm × 1.6 cm × 1.9 cm. Points: 3. TIRADS 3. (C) R lower pole thyroid nodule in longitudinal axis measuring^1^ 1.52 cm × ^2^ 1.8 cm. (D) R lower pole thyroid nodule in transverse axis measuring 3 1 cm. Lower pole, mixed cystic and solid (1), hypoechoic (2), taller-than-wide (3), ill-defined (0), and no calcs (0). Measuring 1.5 cm × 1.8 cm × 1 cm. Points: 6. TIRADS 6. (E) L mid-pole thyroid nodule in longitudinal axis measuring^1^ 1.6 cm × ^2^ 0.8 cm. (F) L mid-pole thyroid nodule in transverse axis measuring ^3^ 1.4 cm. Mid-pole, almost completely solid (2), hypoechoic (2), wider-than-tall (0), ill-defined (0), and no calcs (0). Measuring 1.6 cm × 0.8 cm × 1.4 cm. Points: 4. TI-Rads: 4. (G) L thyroid doppler in longitudinal axis with normal vascularity. (H) R thyroid doppler in longitudinal axis with hypervascularity more than the L side.

**Figure 3:**
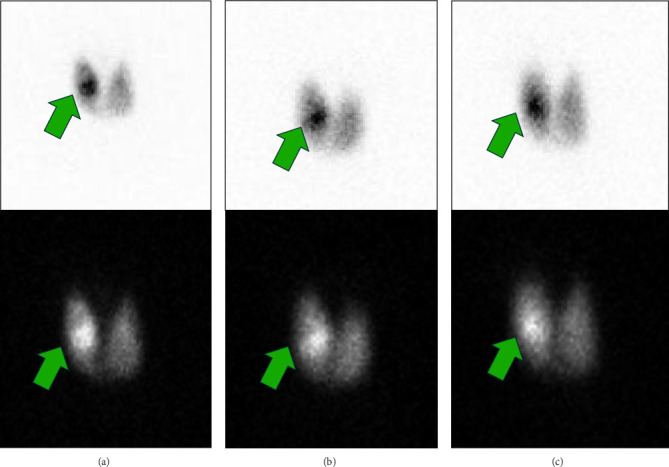
Radioactive iodine thyroid uptake scan. (A) Anterior view, (B) right lateral view, and (C) left lateral view. 213.247 microcurie I-123 sodium iodide orally with 24 h radioactive iodine uptake of 75.1% of the administered dose (normal range 10%–35%) in the whole thyroid gland (comptabile with Graves' disease). Increased radiotracer uptake within sonographically demonstrated right midpole thyroid nodule consistent with hyperfunctioning thyroid nodule (green arrow).

Patient responded well to medical treatment and was discharged during follow-up, the methimazole dose gradually reduced, and GD antibodies declined (Table 1). Repeat thyroid US after 9 months revealed changes in the previously detected nodules (R mid-pole nodule became 2.9 cm with the same TIRADS 3 classification and L mid-pole thyroid nodule became 1.6 cm, and classified as TIRADS 4) and identified a new R lower pole thyroid nodule measured 1.8 cm, described as hypoechoic, and taller than wide and classified as TIRADS 6 (Figure 2). A 24-h radioactive sodium iodide (RASI) thyroid uptake scan with 213.247 microcurie Iodine-123 showed an increase uptake 75.1% (normal range 10%–35%) and AFTN in R mid-pole (TIRADS 3 nodule) and the other two nodules (TIRADS 4 and 6) in the corresponding US had the same high uptake of the surrounding thyroid tissue (warm nodules) with no cold nodules (Figure 3).

**Figure 4:**
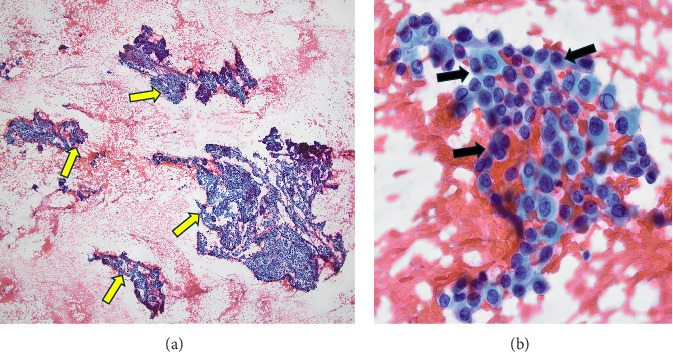
Right lower Thyroid nodule fine-needle aspiration biopsy shows malignant papillary thyroid cancer cells; Bethesda VI. (A) benign thyroid cells and hypercellular malignant cells with papillary architecture (yellow arrow) (H&E low power). (B) Right lower pole biopsy with malignant thyroid cells with enlarged and elongated nuclei (black arrow) (H&E high power).

Thyroid fine-needle aspiration (FNA) biopsy was done to the three nodules and revealed benign Bethesda II results for the R mid-pole (AFTN) and L mid-pole nodules, while the R lower pole nodule (the TIRADS 6; warm nodule) showed papillary thyroid carcinoma (PTC) cells (Bethesda VI) (Figure 4). Neck US did not demonstrate suspicious lymph nodes.

**Figure 5:**
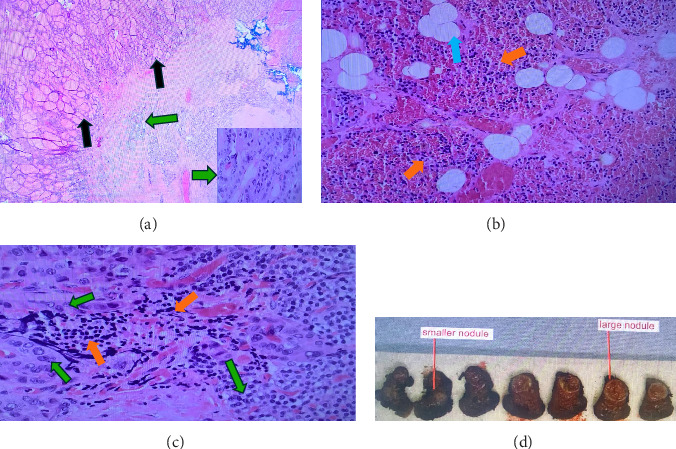
Right hemithyroidectomy gross and microscopic pathology. (A) Right hemithyroidectomy shows benign cells with follicular arrangement (black arrow) and malignant thyroid cells with hypercellularity and papillary architecture (green arrow). H&E low power and high power of malignant cells in the right side. (B) Intrathyroidal parathyroid tissue with fat cells, adipose tissue (blue arrow), and chief cells (orange arrow). (C) Overlap between malignant thyroid cells (green arrow) and parathyroid tissue (orange arrow) indicating intrathyroidal parathyroid tissue (H&E high power). (D) Gross pathology of the right hemithyroidectomy with large mid-pole nodule, the hyperfunctioning nodule, and smaller lower pole nodule which harbors the papillary thyroid cancer.

R hemithyroidectomy was performed with removal of one level VI lymph node and reimplantation of the R inferior parathyroid gland into the sternohyoid muscle. Surgery was complicated by R recurrent laryngeal nerve (RLN) injury. Pathology examination revealed a 0.9 cm× 0.8 cm unifocal classic differentiated PTC, staged pT1aN0a, mitoses <3 mm/2 mm^2^ with extension to the anterior surgical margin and parathyroid gland. No perineural, lymphatic, or vascular invasion, and no lymph node metastasis were noted in the resected tissue. Further pathology review has found intrathyroidal parathyroid tissue with minimal PTC spread to it (Figure 5). Multidisciplinary team discussion recommended RASI therapy due to positive anterior surgical margin and to control GD, as well with plan of RLN primary repair.

## 3. Discussion

Thyroid nodules are common with GD affect up to 44% of cases in some studies [3]. The coexistence of AFTN with GD is rare and known as MLS. There are three types of MLS: Type 1 characterized by one AFTN, Type 2 characterized by two or more AFTN, whereas Type 3 characterized by combination of AFTN and hypofunctioning (cold) nodules [4]. Our case has MLS Type 2 with single AFTN in the R mid-pole and two warm nodules. Up to 10%–15% of nodules in GD are malignant, predominantly PTC [5]. One study found that 20% of surgically treated GD patients had TC, particularly unifocal microcarcinomas in middle-aged women like our case [6]. A Taiwanese database study identified an increased risk of TC (HR: 10.4) and breast cancer (HR: 1.58) in patients with GD [7].

Our patient was diagnosed with GD in the setting of thyroid storm with two TIRADS 3 nodules. Following several months of medical therapy and achievement of euthyroidism, repeat thyroid US demonstrated changes in these nodules; R thyroid nodule changed from 3.3 cm to 2.9 cm in the largest dimension and L thyroid nodule changed from 2.5 cm to 1.6 cm in the largest dimension and change in the consistency from isoechoic to hypoechoic which, in turn, changed the TIRADS classification from 3 to 4. Surprisingly, a new R thyroid nodule with the highest risk descriptive criteria and classification of TIRADS 6 was identified. The discrepancy between the two thyroid US could be explained by GD induced parenchymatous hyperplasia and hypertrophy which resolved following the control of hyperthyroidism leading to decrease in size and change of consistency of previously detected nodules and unmasking a previously obscured high-risk nodule. The FNA biopsy revealed Bethesda VI with PTC cells in the R nodule (TIRADS 6) and the other two nodules were benign; Bethesda II. Our initial plan was total thyroidectomy to control both the TC and GD. However, due to injury to the R RLN, only a R hemithyroidectomy was performed. Lymph node dissection was not performed, as neck US did not show suspicious lymph nodes. Pathology demonstrated stage I 0.9 cm differentiated thyroid cancer cells with classic papillary subtype, positive anterior surgical margin, and extension to the parathyroid tissue, however, further review revealed the presence of intrathyroidal parathyroid tissue with no separating capsule between thyroid and parathyroid tissue and excluded extrathyroidal extension. Given the positive surgical margin and the risk of L RLN injury as our patient had postoperative R vocal cord paralysis, RASI therapy was preferred over completion thyroidectomy. This decision was supported by iodine-avid thyroid tissue and the unproven malignancy in the L nodule and aimed to ablate the remnant thyroid tissue and control both TC and GD. Thyroid hormone replacement is planned post-RASI therapy.

A case of MLS and severe ophthalmopathy was reported and emphasized the need for surgical intervention due to the diagnostic complexity and potential for malignancy [8]. They support the role of thorough nuclear imaging and histological confirmation in MLS diagnosis and management [8]. Additionally, another case of MLS with concomitant PTC in hyperfunctioning nodule was described [9]. This parallels our case, where the TIRADS 6 “warm” nodule turned out to be malignant despite its functional appearance on the uptake scan. Another layer of MLS complexity was defined with a novel variant: MLS with thyroid isthmus agenesis, an extremely rare anatomical presentation [10]. This case emphasized the value of comprehensive pre-operative imaging and the potential for unusual congenital anomalies in MLS [10]. From a long-term perspective, a case of recurrent goiter and MLS presentation 27 years after initial thyroid surgery was described, highlighting the importance of lifelong monitoring even in previously treated or “cured” cases [11].

Several hypotheses have been implemented for the association between GD and TC as oxidative DNA damage by inflammatory cytokines and fibroblast with autoimmune disorders that can induce malignancy [12]; however, this hypothesis is not unique for GD as it can happen with other autoimmune disorders as Hashimoto's thyroiditis but that can be relevant to our case as she has positive GD and thyroid peroxidase antibodies. The role of GD antibodies is controversial; some studies did not find evidence of association while others found that patients with high antibodies titer have low risk of TC [13]. Other hypotheses included the increase expression of the programed death ligand-1 and Fork head box P3 genes, and microRNAs in GD and their role of TC development [14]. Moreover, larger and multiple thyroid nodules in GD patients have been associated with an increased TC risk in another study [15].

The prognosis of TC with GD is variable in literature from no difference in outcome [16] to aggressive course with high recurrence risk and metastasis versus better prognosis and longer survival [11]. A systematic umbrella review and meta-analysis examining the association between GD and TC risk, and prognosis found strong evidence linking TC with increased mortality risk, and moderate evidence indicating a higher recurrence risk in patients with GD than in those without GD [14].

Similar to the variability in prognosis, the management of TC in patients with GD is not standardized in clinical guidelines. Some studies advocate for total thyroidectomy with lymph node dissection and radioactive iodine therapy due to the potential for an aggressive disease course and poorer prognosis. In contrast, others argue against intensive management, citing no significant impact on outcomes or even a possibility of better survival [14, 17].

## 4. Conclusion

MLS should be considered in the differential diagnosis of GD. Thorough clinical assessment of GD for potential thyroid nodules is crucial, as routine thyroid US is not typically recommended in GD cases. Further evaluation of thyroid nodules by nuclear imaging and FNA biopsy is essential, particularly for high-risk nodules due to the increasing prevalence of TC with GD and thyroid nodules. The management and prognosis of TC in GD remain inconsistent, emphasizing the need for individualized treatment strategies and further research to establish standardized clinical guidelines.

## Figures and Tables

**Table 1:** Thyroid hormones and antibodies were monitored from the initial presentation through follow-up visits, with corresponding adjustments in management.

Thyroid hormones and antibodies	Initial labs (thyroid storm)	5 Days later (on discharge)	1 Month follow-up	3 Months follow-up	6 Months follow-up	1 Year follow-up (days before R hemithyroidectomy)	13 Months follow-up (1 month after R hemithyroidectomy)
TSH (normal 0.35–4 mcIU/mL)	<0.01	<0.01	<0.01	2.63	0.96	1.08	3.85

Free thyroxine (T4) (normal 0.6–1.7 ng/dL)	>6	5.50	3.30	0.33	0.99	0.95	0.80

Total triiodothyronine (T3) (normal 80–178 ng/dL)	463	—	167	130	142	125	93

TSI Ab (normal less than 0.54 IU/L)	29.40	—	—	15.1	8.41	4.24	1.10

TRAb (normal less than 1.75 IU/L)	33.50	—	—	22.60	11.51	5.26	2.15

TPO Ab (Normal less than 9 IU/mL)	57.10	—	—	—	—	—	—

Management	- Methimazole 20 mg every 6 h - potassium iodide five drops every 6 h (50 mg/drop, 0.05 mL); started 2 h after methimazole - esmolol infusion then Propranolol 60 mg every 8 h - hydrocortisone 50 mg every 8 h	- Methimazole 20 mg every 6 h - propranolol 60 mg every 8 h	- Methimazole 20 mg every 12 h - propranolol was stopped	- Methimazole 10 mg daily	- Methimazole 7.5 mg daily	- Methimazole 7.5 mg/day	- Methimazole was stopped - planning for RASI therapy, followed by thyroid hormone replacement

*Note*: Patient had primary hyperthyroidism with positive TSI and TRAB consistent with Graves' disease. TPO antibodies were positive. She presented with thyroid storm and managed medically as outlined till the discharge. Clinical and biochemical euthyroidism were achieved.

Abbreviations: Ab, antibody; h, hours; IU/mL, international unit per milliliter; mcIU/mL, micro-international units per milliliter; mg, milligram; ng/dL, nanogram per deciliter; ng/mL, nanogram per milliliter; R, right; RASI, radioactive sodium iodide; TPO, thyroid peroxidase; TRAB, thyroid hormone receptor antibody; TSH, thyroid stimulating hormone; TSI, thyroid stimulating immunoglobulin.

**Table 2:** Laboratory workup on the initial presentation and during the follow up.

Laboratory workup	Initial labs on admission	3 Months follow-up	6 Months follow-up	1 Year follow-up (days before R hemithyroidectomy)	13 Months follow-up (1 month after R hemithyroidectomy)
AST/ALT (normal 10–40 and 7–50 U/L, respectively)	29/54	10/20	12/23	11/11	11/13
GFR mL/min	138	149	130	140	155
Creatinine (normal 0.4–1.2 mg/dL)	0.38	0.4	0.3	—	—
Serum calcium (normal 8.6–10.6 mg/dL)	9	9.10	8.89	10	9.95
Parathyroid hormone (normal 12–88 pg/mL)	49	—	—	—	51
Hemoglobin (normal 11.7–16 g/dL)	12.9	13	12.5	11	12.9
White blood cells (normal 3.9–12 k/UL)	10.6	9.5	8.10	10	10.5
Platelets (normal 150–450 k/UL)	325	350	400	390	330

Abbreviations: ALT, alanine transferase; AST, aspartate transferase; g/dL, gram per deciliter; GFR, glomerular filtration rate; k/μL, kilo per microliter; mg/dL, milligram per decliter; mL/min, mililiter per minute; N, normal; pg/mL, picogram per mililiter; R, right; U/L, unit/liter.

## Data Availability

All the data generated or analyzed during this study are included in this published article. Further inquiries can be directed to the corresponding author.
